# P07-03 The characteristics of the medical prescription for adapted physical activity among the French Health System, experience of the Grand Est region

**DOI:** 10.1093/eurpub/ckac095.103

**Published:** 2022-08-29

**Authors:** Bruno Chenuel

**Affiliations:** 1 Center of Sports Medicine and Adapted Physical Activity, University Hospital of Nancy, Vandoeuvre-lès-Nancy, France; 2 DevAH -Department of Physiology, University of Lorraine, Vandoeuvre-lès-Nancy, France

**Keywords:** Adapted Physical Activity, Exercise, Therapeutic Education, Non-drug prescription, French health system

## Abstract

**Issue/problem:**

The law of modernization of the French health system promoted in 2016 have authorized any attending physician to prescribe a program of physical activity suitable for patients with a chronic disease. The health benefits of active living habits are now well known but too few physicians have seized this prescription of Adapted Physical Activity (APA).

**Description of the problem:**

APA teachers have come to complement the health professions to deliver APA, and sports clubs have improved their organization to welcome patients who have adequate physical condition. Regional systems have been deployed to receive patients, using such resources. The ‘Médicosport Santé’ guide provides relevant guidance for patients according to physical and/or sporting activities (PSA) and diseases. The medical prescription for APA must therefore become a practical reality for chronic disease patients, guaranteeing a lasting change in lifestyle through more active behavior.

**Experience from the Grand Est country:**

The commitment of certain French Regional Health Agencies, has led certain regions to offer regional systems, as the “Prescri'mouv” plan in the Grand Est country, allowing a step forward to best support the patient from the medical prescription to regular and lasting practice. After medical prescription, patients are directed to an APA professional or physiotherapist, for an initial evaluation and orientation towards one of the three types of care: autonomous practice, labeled structure, specific support.

**Lessons:**

The main objective of APA is to fight against a sedentary lifestyle. With an adequate physical condition, physical activities and sports are to be considered in order to help patients with chronic disease, sources of pleasure and lasting health benefits. New actors have come to strengthen health professionals, and through ‘Sport Santé’ concept, sports clubs have organized themselves to welcome patients.

**Main messages:**

Adapted physical activity is the keystone in the fight against sedentary lifestyle.

New players have come to strengthen health professionals, sports clubs have organized themselves to welcome of and best support patients with chronic disease.

The challenge is for physicians to take hold of this medical prescription for APA, by directing patients towards more active lifestyle habits.

